# A useful approach to total analysis of RISC-associated RNA

**DOI:** 10.1186/1756-0500-2-169

**Published:** 2009-08-26

**Authors:** Yukinobu Hayashida, Takahiro Nishibu, Kunio Inoue, Tsutomu Kurokawa

**Affiliations:** 1 Genome Research Laboratories, Wako Pure Chemical Industries, Ltd. Takada 6-1, Amagasaki, Hyogo 661-0963, Japan; 2 Department of Biology, Graduate School of Science, Kobe University, 1-1 Rokkodaicho, Nadaku, Kobe 657-8501, Japan

## Abstract

**Background:**

Identifying the endogenous RNA induced silencing complex(RISC)-associated RNAs is essential for understanding the cellular regulatory networks by miRNAs. Recently, isolation of RISC-associated mRNAs using antibody was reported, but their method needs a large amount of initial materials. We tried to improve the protocol and constructed an efficient and convenient system for analyzing miRNA and mRNA contents in RISC.

**Findings:**

With our protocol, it is possible to clone both miRNAs and mRNAs from the endogenous RISC-associated RNAs immunoprecipitated from less than 10^7 ^cells, and we show the ability of our system to isolate the particular target mRNAs for a specific miRNA from the RISC-associated mRNAs using well-characterized miR-122 as an example. After introduction of miR-122 into HepG2 cells, we found several cDNA clones that have miR-122 target sequences. Four of these clones that were concentrated in RISC but decreased in total RNA fraction are expected to be miR-122 target candidates. Interestingly, we found substantial amounts of Alu-related sequences, including both free Alu RNA and Alu-embedded mRNA, which might be one of the general targets for miRNA, in the cDNA clones from the RISC-associated mRNAs.

**Conclusion:**

Our method thus enables us to examine not only dynamic changes in miRNA and mRNA contents in RISC but also the relationship of miRNA and target mRNA. We believe that our method can contribute to understanding cellular regulatory networks by miRNAs.

## Background

MicroRNAs (miRNAs) are approximately 22-nucleotide endogenous non-coding RNAs that play important roles in post-transcriptional regulation of gene expression by base-pairing to their target mRNAs [1]. After being transcribed and processed, mature miRNAs are incorporated into the Argonaute protein family, the core component of the RNA-induced silencing complex (RISC), for targeting mRNAs based on sequence complementation in 3'UTRs [2-4]. In humans, the Argonaute family consists of eight members, divided into the Ago subfamily (Ago1-Ago4) and Piwi subfamily (PIWIL1-PIWIL4) [5]. Although all four Ago subfamily members have been implicated in translational inhibition of mRNA [6], only one Ago protein, Ago2, possesses intrinsic endonuclease activity. Experiments in mice and human cell lines have shown that Ago2 is the central RISC component, capable of cleaving target mRNA when perfect complementarity with it exists [7-12].

Identifying the target mRNA against miRNA is essential to understand cellular regulatory networks by miRNAs. Due to the low complementarity between a miRNA and its target mRNAs, only a few mammalian target mRNAs have been identified. Combinations of biochemical and computational approaches have been started to elucidate how mRNA targets are specifically recognized by miRNAs. Among biochemical approaches, recovery of miRNA from RISC using antibody [13-16] has already been reported, and recently that of RISC-associated mRNA from the immunoprecipitates was also reported [17-21]. However, isolation of RISC-associated mRNA has mainly been performed using exogenously introduced Tagged-Ago2 or GW182 in combination with antibodies to the Tag [18-21], and the protocol needs improvement for the efficient analysis of the RISC-associated mRNA without using large amounts of initial materials [17].

## Methods

### Cell culture and transfection

HeLa, HEK293, and HepG2 cells were grown in DMEM supplemented with 10% fetal calf serum. THP-1 and P388D1 cells were grown in RPMI1640 supplemented with 10% fetal calf serum. Transfection of HepG2 cells was performed using Lipofectamine RNAi max reagent (Invitrogen). A total of 5 × 10^6 ^cultured cells were transfected with 600 pmol miR-122 siRNA (5'-UGGAGUGUGACAAUGGUGUUUGU-3', 5'-AAACACCAUUGUCACACUCCAUA-3') or control siRNA firefly luciferase GL3 (Nippon Gene) according to the manufacturer's instructions. After 24 hours, transfected cells were collected.

### Immunoprecipitation and RNA purification

Cultured cells (5 × 10^6^~1 × 10^7 ^cells) were collected by trypsinization and washed with PBS(-). Cells were incubated in 1 ml of lysis buffer (20 mM Tris pH7.4, 2.5 mM MgCl_2_, 200 mM NaCl, 0.05% NP40) for 10 min on ice, and the cell lysates were cleared by centrifugation at 20000 ×g for 20 min at 4°C. Twenty μl of ProteinG coupling beads (Dynal) bound with 5 μg of anti-human Ago2 antibody 4G8 (Wako) was added to cell lysate and mixed by rotation for 2 hours at 4°C. The beads were then washed three times with 1 ml lysis buffer, and bound Ago2-RNA complex were eluted with 0.5% SDS solution. Eluted complex was extracted with phenol/chloroform, and the RNA was precipitated with ethanol.

Total RNA from cultured cells was extracted using ISOGEN (Nippon Gene). A small RNA fraction (< 200 nt) was obtained using the *mir*Vana™ miRNA Isolation Kit (Ambion). Small RNA 20–24 nt long was purified from the small RNA fraction by denatured 15% polyacrylamide gel electrophoresis, treated with phenol/chloroform/isoamyl alcohol (25:24:1), and ethanol-precipitated.

### Gel electrophoresis of immunoprecipitates

The immunoprecipitate from 5 × 10^6 ^cells was loaded onto 10–20% SDS-polyacrylamide gel. After electrophoresis, the protein was stained with a Wako Silver staining kit (Wako). Western blot examination was performed using anti-human Ago2 antibody 4G8.

The immunopurified RNA derived from 5 × 10^6 ^cells was loaded onto 10% polyacrylamide TBE-Urea gel and run in 0.5 × TBE at 10 mA for 50 min. After electrophoresis, the gel was silver stained with Clear stain Ag (Nippon Gene) to check amounts of small RNA.

### cDNA cloning of small RNA

The RNA was dephosphorylated with 1 unit of shrimp alkaline phosphatase (USB) for 30 min at 37°C in 50 mM Hepes (pH7.3), 5 mM MgCl_2_, and 10 mM KCl. Then a 5' phosphate and 3' dideoxy modified 3' RNA adaptor (5'-prArArGrArArGrCUrGrGrCrGUrCrArAUrGUddC-3') was ligated with 10 units of *ThermoPhage*™ single-stranded DNA ligase (PROKARIA) for 30 min at 60°C in 25 mM Hepes (pH7.3), 2.5 mM MgCl_2_, 5 mM KCl, 0.05 mM ATP, 1 mM dithiothreitol (DTT), 25 ng/μl BSA. The reaction liquid was treated with phenol/chloroform/isoamyl alcohol, and ethanol-precipitated. The 3' adaptor ligated RNA and 2.5 μM of a first-strand primer (5'-GACATTGACGCCAGCTTCTT-3') with sequence complementary to the ligated 3' RNA adaptor were mixed and the cDNA was synthesized with 200 units of ReveScript IV reverse transcriptase (Wako) for 30 min at 42°C in the manufacturer's 1 × reaction buffer (50 mM Tris-HCl, pH8.3, 75 mM KCl, 3 mM MgCl_2_, 5 mM DTT, 2 mM dNTPs with 20 units of Ribonuclease Inhibitor, Super (Wako)). The mixture was incubated for 30 min at 65°C in 167 mM EDTA and 53 mM NaOH to hydrolyze the RNA template. Then the hydrolyzed reaction mixture was neutralized with 20 mM Tris-HCl, pH 7.5 and treated with phenol/chloroform/isoamyl alcohol, and ethanol-precipitated. The remaining single-stranded cDNA was dephosphorylated with shrimp alkaline phosphatase and ligated to the 5' phosphated and 3' dideoxy modified 5' adaptor (5'-pAAGGCTCAGTCTCGGGATAddC-3') with *ThermoPhage*™ single-stranded DNA ligase. The ligated cDNA was amplified by 2-step PCR with 2.5 units of HotGoldstar™ DNA polymerase (Eurogentec) in the manufacturer's 1 × reaction buffer with 0.25 mM dNTPs, 5 μM of 5' PCR primer (5'-GTATCCCGAGACTGAGCC-3'), and 5 μM of 3' PCR primer (5'-GACATTGACGCCAGCTTC-3') corresponding to 5' adaptor and 3' adaptor sequence, respectively. The reaction was performed at 95°C for 10 min, 95°C for 20 sec, 56°C for 10 sec, and 72°C for 10 sec for 15 cycles, and 7(EtBr) staining. The purified PCR product was amplified by a second PCR with the 2°C for 1 min. After the first 15 cycles, products were loaded on a 15% native polyacrylamide gel electrophoresis and 60–64 bp fragments were excised after ethidium bromide same conditions as the first PCR, and the product was purified by gel electrophoresis. The product was ligated to 20 ng of pGEM T Easy Vector (Promega) with a DNA Ligation Kit Mighty Mix (Takara Bio) and transfected into DH5α. Plasmid DNA was prepared with the QuickGene Plasmid Kit S (FUJIFILM) and sequenced with the BigDye^® ^Terminator Cycle Sequencing Kit (Applied Biosystems). Each miRNA was identified using the BLAST homology search program .

### cDNA cloning of anti-Ago2- immunoprecipitated mRNA

The first-strand cDNA was synthesized with 200 units of ReveScript IV reverse transcriptase for 10 min at 42°C in the manufacturer's buffer (50 mM Tris-HCl, pH8.3, 75 mM KCl, 3 mM MgCl_2_, 5 mM DTT, 2 mM dNTPs with 20 units of Ribonuclease Inhibitor, Super) using oligo-dT(20) primer (5'-GACCATATGACGAGATCCGAGCTTCTTTTTTTTTTTTTTTTTTTT-3') with the sequence for PCR amplification. Then the mixture was incubated for 30 min at 65°C in 167 mM EDTA and 53 mM NaOH to hydrolyze RNA template. The hydrolyzed mixture was neutralized with 20 mM Tris-HCl, pH 7.5 and purified with the Invisorb^® ^Spin PCRapid Kit (Invitek). Then the second-strand cDNA was synthesized using 4 μM random primers (5'-CATGGTATCGACGAGACTGAGGCTGNNNNNNNNNB-3', N = A, C, G, T, B = C, G, T) containing the sequence for subsequent PCR amplification with 2.5 units of HotGoldstar™ DNA polymerase in the manufacturer's reaction buffer with 0.2 mM dNTPs. The reaction was performed at 95°C for 10 min, 30°C for 1 min, 40°C for 30 sec, 50°C for 30 sec, 60°C for 30 sec, 72°C for 2 min, and 95°C for 1 min, followed by chilling on ice for 1 min, and cDNA was then purified with the Invisorb^® ^Spin PCRapid Kit. The double-stranded cDNA was amplified by PCR with 2.5 units of HotGoldstar™ DNA polymerase in the manufacturer's reaction buffer with 0.2 mM dNTPs and 4 μM each of the 5' PCR primer (5'-CATGGTATCGACGAGACTGAG-3') and 3' PCR primer (5'-GACCATATGACGAGATCCGAG-3'). The reaction was performed at 95°C for 10 min, 95°C for 20 sec, 60°C for 10 sec, and 72°C for 30 sec for 24 cycles, and 72°C for 2 min. The amplified cDNA was purified with the Invisorb^® ^Spin PCRapid Kit. The size distribution of the cDNA was analyzed with the Bioanalyzer DNA 1000 Kit (Agilent Technologies). The residue of purified PCR product was ligated to 20 ng of pGEM T Easy Vector with DNA Ligation Kit Mighty Mix for 1 h at 16°C to construct a cDNA library and transformed into DH5α. Plasmid DNA was prepared with the QuickGene Plasmid Kit S and sequenced with the BigDye^® ^Terminator Cycle Sequencing Kit. The identification of mRNA species corresponding to each cDNA sequence was performed using the BLAST homology search program. To detect microRNA target sites in isolated mRNA, the microRNA target prediction search program TargetScan 4.2  was used. In addition, mRNA expression data for cells and tissues were searched using the microarray database RefExA . Free Alu RNA or Alu RNA embedded mRNA was predicted by a homology search against three types of human primary Alu transcript (GeneBank accession Nos. U67811, U67812, and U67813) using GENETYX^® ^(GENETYX) gene analysis software, and molecules with greater than 60% homology were classified as Alu RNA-related. 'Hit in genome' demonstrates the sequences that were not categorized by BLAST search.

### Quantitative-PCR

Reverse transcription of mRNA was performed using the SuperScript VILO cDNA Synthesis Kit (Invitrogen) following the manufacturer's instructions. Immunopurified RNA (derived from all transfected cells) and 1 μg of total RNA were used for reverse transcription. Quantitative-PCR of mRNA was performed using gene specific primer pairs and 2 × Power SYBR Master Mix (Applied Biosystems) following the manufacturer's instructions. Quantitative-PCR were performed on the ABI 7500 real-time PCR system.

## Results and Discussion

### Isolation of endogenous Ago2 and miRNA complex

For the immunoprecipitation of Ago2 protein, we used an anti-human Ago2 antibody 4G8, which has been described for its immunoprecipitating activity and specificity [13,22]. SDS-PAGE analysis of the immunoprecipitates from human cell line extracts of HeLa, HepG2, HEK293 and THP-1 but not of mouse cell line P388D1 yielded a clear Ago2 protein band of approximately 100 kDa. Also, PAGE analysis revealed a highly purified RNA band of approximately 22 nucleotides long in the immunoprecipitates of all human cell lines tested (Additional file 1). In addition, we also ascertained this procedure can be applicable to the tissue extract. To profile the immunopurified RNA, we performed a microarray analysis using the μParaflo^® ^microfluidics chip (LC Sciences) with a complementary sequence of human 474 miRNAs on it (miRBase release 9.0) and compared the profile with that of total RNA. The miRNA profile of HeLa cell immunopurified RNA exhibited almost the same pattern with that of total RNA (Additional file 2) suggesting that Ago2-immunopurification could trap almost all expressed miRNA species.

### Construction of a simple and efficient method for cloning of miRNA

**Figure 1:**
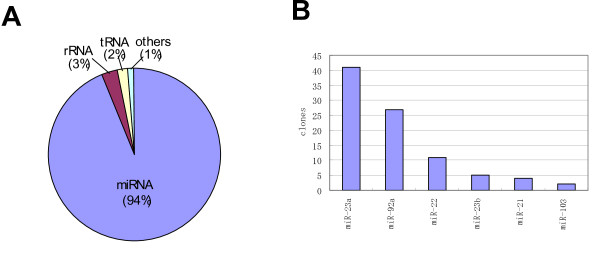
**Categories of cDNA clones derived from immunoprecipitated small RNA of HeLa cells**. **A: **Composition of small RNA cDNA clones. The composition of cDNA clones is shown in a circle graph. Almost all of the recovered cDNA was miRNA (94%), while the remainder was rRNA and tRNA clones (3% and 2%). 'Others' indicates the cDNA clones with hits in the genomic sequence. **B: **Contents of miRNA clones. Each miRNA clone was deduced by BLAST search. The y-axis indicates number of clones of each miRNA species.

The cDNA of the isolated small RNA was synthesized by improved adaptor ligation protocol see Methods section. We utilized the unique features of *ThermoPhage*™ single-stranded DNA ligase, which has ligase activities for both single-stranded RNA and DNA and can be used at 60°C [23]; this ligase was used for all of the adaptor ligation. High temperature may be advantageous for the ligation of nucleotides with tight tertiary structures. Using this method, cDNA libraries for the Ago2-immunoprecipitated RNA were produced, and the sequences of 96 clones were analyzed. The contents of the clones are shown in Figure 1A. Although significant amount of contamination by junk cDNA fragments derived from adaptor or primer sequences has been reported in other cloning methods [24], no similar contamination was found in the library made with our method. Moreover, marked reduction in the number of rRNA and tRNA clones was observed, compared to the other systems [24]. The profiles of cloned miRNA are listed in Figure 1B. Though the number of clones examined here may still be insufficient, these results suggest the usefulness of our Ago2-immunoprecipitation/cloning system of Ago2-RISC-associated miRNAs.

### Cloning of antibody-isolated mRNA

Since a significant amount of mRNA is known to be immunoprecipitated with RISC-associated RNA, we developed a cloning system for the trapped mRNA. Because the mRNA content in the immunoprecipitates from 10^7 ^cells was small and varied in size, we included a PCR amplification step in the cloning of immunoprecipitated mRNAs. To avoid PCR-derived bias due to the variation in the length of mRNA for cloning efficiency, we did not aim to synthesize the full-length cDNA and used random primers for the second-strand cDNA synthesis (Additional file 3). The human mRNA database is now almost completed and the sequence information of 3' short cDNA fragments is sufficient to identify each mRNA species. Independence of the length and terminal cap structure of template RNA may be suitable for comprehensive profiling of mRNAs. We tested the cloning efficiency of three mRNA species with different lengths (albumin: 2122b, beta-actin: 1874b, GAPDH: 1380b). We mixed them in equal amounts and confirmed nearly equal ratios of cloning for these input mRNAs (15, 19, and 14 clones from total 48 clones, respectively). With this protocol, cDNA cloning of immunoprecipitated mRNA could be achieved with relatively small amount of RNA prepared from 1 × 10^7 ^cells.

**Figure 2:**
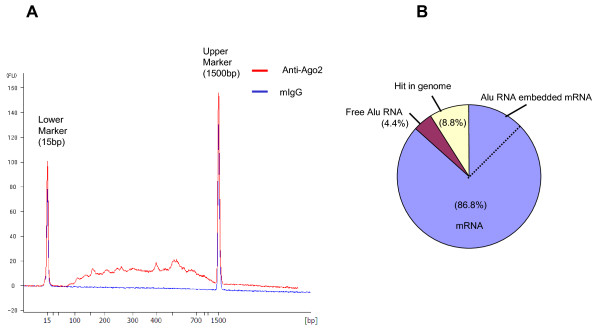
**Size distribution of synthesized cDNA and categories of cDNAs isolated from HeLa cells**. **A: **Size distribution pattern of synthesized cDNA. The amount and size distribution of cDNA were determined with the Bioanalyzer DNA 1000 Kit. The red line indicates cDNA synthesized from anti-Ago2 immunoprecipitates, while the blue line indicates that from control mouse IgG. **B: **Composition of isolated cDNA clones. The sequences of 91 clones were examined by BLAST search and categorized as mRNA, Alu RNA embedded mRNA, free Alu RNA, or hits in the genome. The percentage recovery of each categorized cDNA clone is shown. Alu RNA embedded mRNA (14.3%, not indicated in the figure) is included in the mRNA area (total 80%) and separated by a broken line.

A cDNA library for HeLa cell immunopurified mRNA was constructed using this protocol. After the PCR amplification step, quantity and molecular size of the synthesized cDNAs were analyzed with the Bioanalyzer DNA 1000 Kit. The size distribution of synthesized cDNAs is relatively small with the size from 100 to 1,000 bp (Figure 2A). cDNA synthesized from a control mouse IgG-immunoprecipitated fraction exhibited no DNA bands indicating that the cDNA could be synthesized specifically from anti-Ago2 antibody-trapped mRNA.

We picked 91 clones and analyzed their sequences. The profile of the cDNA clones deduced by BLAST search is shown in Figure 2B, and all isolated mRNA clones are listed in Additional file 4. Of the 91 clones, 79 clones (86.8%) were identified as mRNA, and the average length of inserted cDNA in the 91 clones was calculated to be 244.8 bp including a poly(A) tail of 31.5 bp. In the course of this analysis, we noticed that the cloned cDNAs contained a relatively high percentage of Alu-related sequences. Of the mRNA clones, about 16.5% (13/79 clones) of clones harbored an Alu sequence in the 5'- or 3'-UTR sequences, whereas the percentage of Alu-containing mRNA in human mRNA species is reported to be about 5% [25]. Alu elements can be transcribed in two different fashions, yielding 'free Alu RNAs', which are transcribed from their own promoter, and 'embedded Alu RNAs', which are transcribed as a part of coding RNAs [26]. Based on the results from in silico analysis, it was speculated that Alu RNAs embedded in 3'-UTRs are probable miRNA targets [27]. Our findings may be the first to show that mRNA with Alu sequences are concentrated in RISC-associated mRNA, and suggest the regulation of gene expression by miRNA through these sequences. A substantial number of free Alu RNAs (4 clones) was also found in the cDNA prepared from HeLa cell extract (Figure 2B). These free Alu RNA clones exhibited a high degree of homology with each other, though none had the same sequence (Additional file 5). In contrast, no cDNA clone in the control IgG experiment has Alu sequence. In the case of HepG2 cells (described in the later section), about 10% of total cDNA from Ago2-immunoprecipitated mRNA were clones with the free Alu RNA sequence. These data suggest the possible binding of free Alu RNAs to some sort of miRNA.

### Changes in RISC-associated mRNA after miR-122 introduction into HepG2 cells

**Figure 3:**
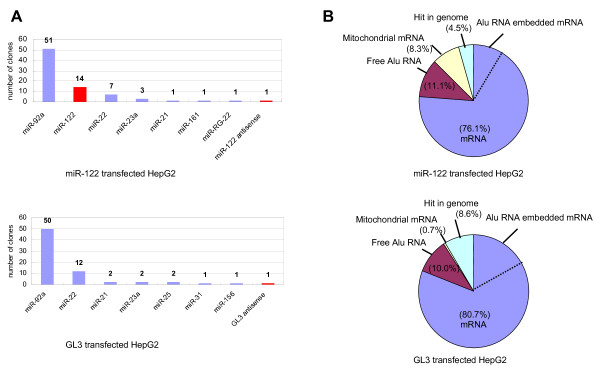
**Contents of miRNA and mRNA cDNA clones obtained from immunoprecipitates of miR-122- and GL3-transfected HepG2 cells**. **A: **Contents of miRNA clones. 89 clones were analyzed for the miR-122-transfectant and 84 for the GL3-transfectant. Red bars show recovered miRNA clones originating from transfected nucleotides (upper figure; miR-122-transfected HepG2 cells, lower figure GL3-transfected HepG2 cells). **B: **Composition of RISC-associated polyA-containing RNA. The isolated cDNA clones were analyzed by BLAST search and classified into categories. The composition of each categorized RNA is shown (Upper figure: miR-122-transfected HepG2 cells, lower figure: GL3-transfected HepG2 cells). Alu RNA embedded mRNA is included in mRNA area. The percentages of Alu RNA embedded mRNA in the miR-122 and the GL3 transfectants were 10% and 19%, respectively.

We tested for our system whether the target mRNAs against a desired miRNA can be screened or not using miR-122 as a model system. The miR-122 is a hepato-specific miRNA, and its down-regulation is reported to be correlated with tumorigenesis in the liver[28]. The human hepatoma cell line HepG2 expresses little miR-122, whereas normal liver cells express it abundantly. We introduced miR-122 duplex or, as a control, firefly luciferase siRNA (GL3) into HepG2 cells transiently and compared the Ago2-associated mRNA profiles of these two types of cells. After transfection of miR-122 duplex, cDNA cloning of miRNA revealed that miR-122 comprised about 16% of immunoprecipitated miRNA (Figure 3A). We did not obtain any GL3 clones, but did obtain a clone of GL3 antisense by GL3 transfection. GL3 was also expected to be cloned like miR-122 so the reason for lack of clones is unclear. After cDNA cloning of mRNA, we picked 289 and 269 clones from miR-122- and GL3-transfected cells respectively, and all identified mRNA clones are listed in Additional file 6.

#### (i) Changes in Alu RNA and mitochondrial mRNA in RISC

The compositions of cDNA isolated from Ago2-associated miR-122- or GL3-transfected HepG2 cells are shown in Figure 3B. Whereas the percentage of free Alu RNA remained constant at about 10% of cDNA, that of Alu-embedded mRNA decreased significantly after miR-122 transfection. These findings may have been obtained because Alu-embedded mRNA does not have target sequences against miR-122 [27]. From the miR-122-transfected cells, substantial amounts of mitochondrial mRNA were cDNA-cloned and almost all of which was *COX3 *(cytochrome c oxidase subunit 3) mRNA. However, since mitochondrial mRNAs are retained inside of the mitochondria in cells, these findings must be artifactual. Since *COX3 *mRNA has two miR-122 target sites, we speculate that during cell lysis, abundant mitochondrial mRNA leaks from the broken mitochondria and reacts with the miR-122.

#### (ii) Possible approach to screening of miR-122 target mRNA

**Figure 4:**
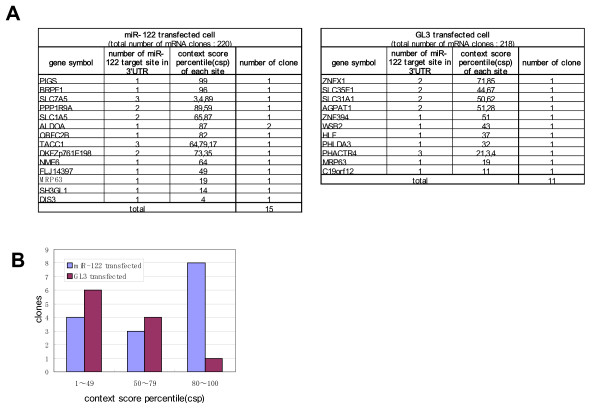
**Selection of target mRNA candidate for miR-122**. **A: **List of cDNA clones with miR-122 seed match sequence in 3'UTR. All isolated clones were searched for the miR-122 target site by the miRNA target prediction program TargetScan 4.2, and the clones exhibiting context score percentiles (csp) are listed in the box. The left box shows the clones isolated from the miR-122-transfectant, and the right box shows those isolated from the GL3-transfectant. **B: **Enrichment of recovered cDNA clone containing miR-122 target site with high csp after miR-122 transfection. The numbers of cDNA clones with csp values for the miR-122 target site categorized as low (1–49), intermediate (50–79), and high (80–100) are shown separately. The blue bar shows the number of clones recovered from the miR-122 transfectant, while the purple bar shows those recovered from the GL3 transfectant.

To search for miR-122 target sequences in mRNA, a miRNA target prediction search program was applied to all isolated clones. In Figure 4A, the cDNA clones containing miR-122 seed match sequences are listed. In the miR-122-transfected HepG2 cells, of 220 mRNA clones, 15 clones had a miR-122 target site, 11 (10 types) of which featured a high context score percentile (>50). In the case of GL3 transfectants, 11 of 217 mRNA clones had a miR-122 target site, 5 of which featured a high context score percentile. As shown in Figure 4B, the clones with high context score percentile for the miR-122 target site were markedly enriched in RISC of miR-122-transfected HepG2 cells.

**Figure 5:**
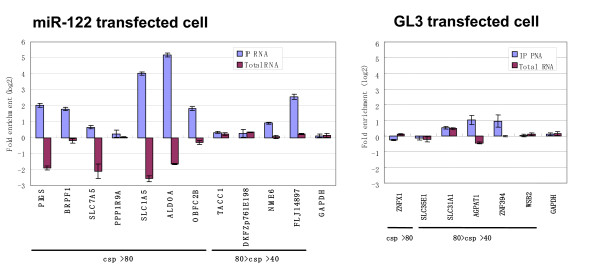
**Changes in mRNA level in total and Ago2-associated RNA after miR-122 transfection**. Seventeen clones with high score percentile for the miR-122 target site (> = 40) were picked, and the mRNA levels in total and immunoprecipitated RNA were quantified by RT-PCR. In the figure, the ratios of the amount of mRNA in the miR-122-transfected cells to that in the GL3-transfected cells are shown. Blue bars indicate the ratio for Ago2-associated RNA, while purple bars show that for total RNA. The left panel shows the clones picked from the miR-122-transfected cells, and the right panel shows the clones picked from the GL3-transfected cells (See Figure 6A). The clones are aligned in order of height of context score percentile from the left, with the ratio for *GAPDH *inserted as a standard.

We selected 17 mRNA clones with high context score percentile (≧ 40) from the miR-122- and GL3-transfectants, and compared the mRNA content in the immunoprecipitate with that in total RNA by real-time PCR. The difference in the RNA content of individual mRNA is shown in Figure 5. Seven mRNA clones selected only from miR-122 transfectant (Figure 5A) were enriched in RISC of the miR-122-transfectants. And since total RNA amounts of four of these clones, *ALDOA *(aldolase A, fructose-bisphosphate), *PIGS *(phosphatidylinositol glycan anchor biosynthesis, class S), *SLC1A5 *(solute carrier family 1 (neutral amino acid transporter), member 5) and *SLC7A5 *(solute carrier family 7 (cationic amino acid transporter y+system), member 5) were decreased in the miR-122 transfectants, these mRNAs are thought to be candidates of miR-122 target. These 4 clones is likely to be down-regulated through interaction with miR-122. In fact, in the RefExA microarray database, these 4 mRNAs are expressed at higher levels in HepG2 cells where less miR-122 is expressed than in normal liver with abundant miR-122. With the exception of *ALDOA*, these mRNAs have not yet been reported as miR-122 targets. To address this issue, other experiments such as translation repression assays may be necessary. Among these candidates, *SLC1A5 *and *SLC7A5 *belong to the amino acid transporters and expressions of these transporters are thought to be necessary for the growth of human liver cancer cells [29], and over-expression of *SLC1A5 *and *SLC7A5 *in liver cancer cells may correlate with a low miR-122 level.

Eventhough the number of clones we examined was very limited and comprised only a portion of libraries we obtained, several miR-122 target candidates could be picked up. We believe that these results show the potential of our protocol for isolating the miRNA targets.

## Competing interests

The authors declare that they have no competing interests.

## Authors' contributions

YH carried out cDNA cloning and database search. TN carried out immunoprecipitation assays and quantitative-PCR assays. KI and TK conceived of the study. All authors contributed to writing of the manuscript.

## Supplementary Material

Additional file 1 **Anti-human Ago2 monoclonal antibody (4G8) specifically immunoprecipitates human Ago2 and associated small RNA**. A: SDS-PAGE pattern of immunoprecipitates. Lysates of human cell lines (HeLa, HepG2, HEK293, and THP-1) and a mouse cell line (P388D1) were immunoprecipitated with anti-hAgo2 (4G8). Proteins were analyzed by SDS-PAGE and subjected to silver staining or western blotting with anti-hAgo2 (4G8). Half of the immunoprecipitated protein prepared from 1 × 10^7 ^cells was loaded onto an SDS-polyacrylamide gel. IgG h.c. and l.c. indicate heavy chain and light chain of the antibody molecule, respectively. B: Recovery of small RNA from immunoprecipitates. Immunoprecipitated small RNAs were analyzed by Urea-PAGE and silver stained. The RNA fraction prepared from half of the immunoprecipitate from 1 × 10^7 ^cells was loaded onto Urea-polyacrylamide gel. Click here for file

Additional file 2 **Comparison of miRNA populations of total RNA and immunoprecipitated RNA in HeLa**. Microarray analysis of miRNA was carried out at LC science inc. Immunopurified RNA and total RNA from HeLa cells were labeled with Cy3 and Cy5. Samples were hybridized to μParaflo^® ^microfluidics chip with each of the detection probes containing a nucleotide sequence of coding segment complementary to human 474 microRNA sequences(miRBase ver.9.0). Click here for file

Additional file 3 **Schematic representation of the cloning protocol for immunoprecipitated mRNA**. The protocol is described in detail in Materials and methods (Additional file 1). Click here for file

Additional file 4 **The list of the cDNA clones derived from mRNA deduced by a BLAST search in HeLa.** Click here for file

Additional file 5 **Homology search for the predicted free Alu RNA clones in HeLa by GENETYX^®^.** Click here for file

Additional file 6 **The list of the cDNA clones derived from mRNA deduced by a BLAST search in miR-122 and GL3 transfected HepG2.** Click here for file
