# Supplementary figures and images for: A useful approach to total analysis of RISC-associated RNA
Source: BMC Res Notes. 2009 Aug 26;2:169. doi: 10.1186/1756-0500-2-169 (PMC2748084; doi:10.1186/1756-0500-2-169)

## Slide 1
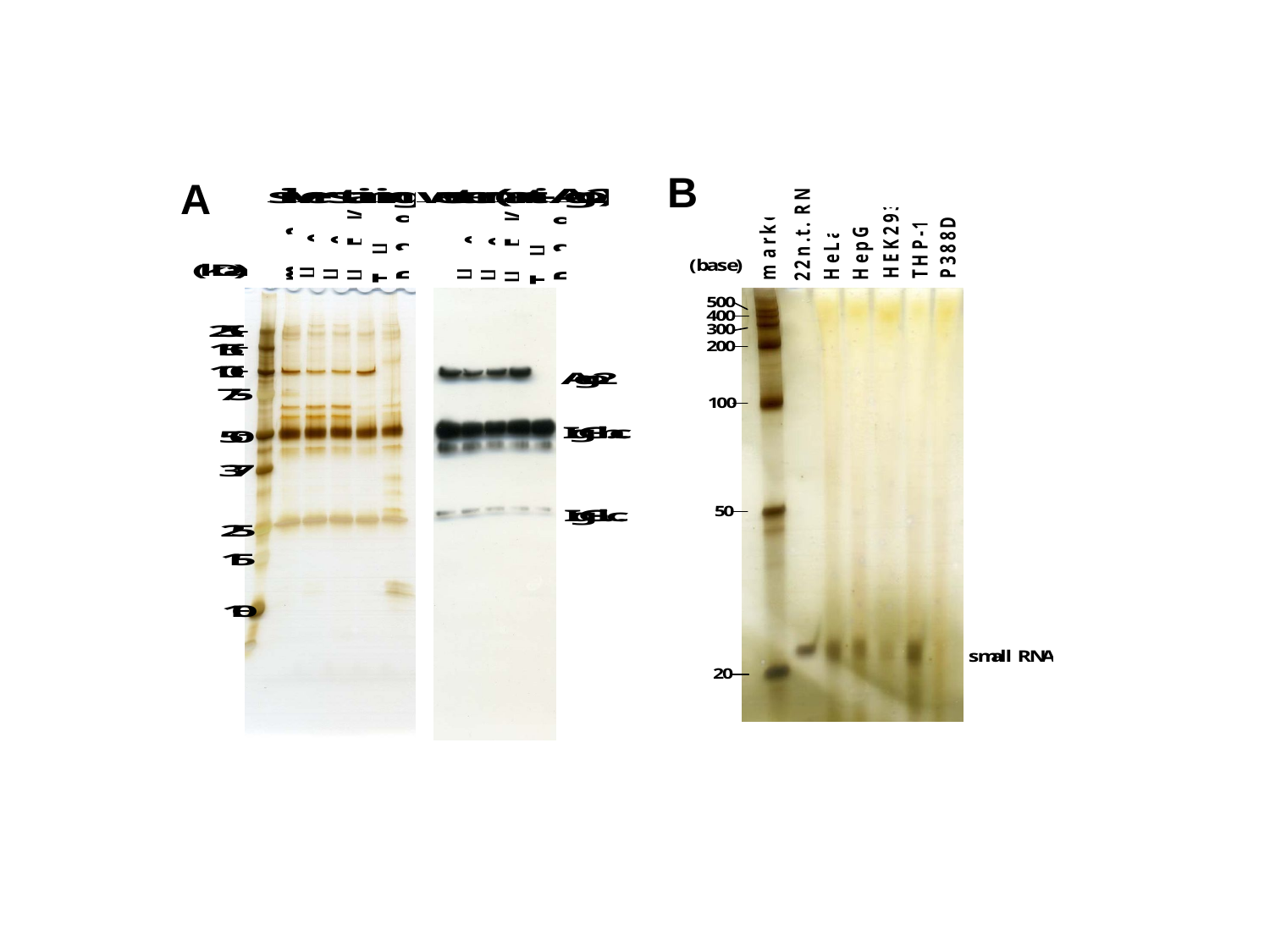

B
A

Supplement: Additional file 1 — Anti-human Ago2 monoclonal antibody (4G8) specifically immunoprecipitates human Ago2 and associated small RNA. A: SDS-PAGE pattern of immunoprecipitates. Lysates of human cell lines (HeLa, HepG2, HEK293, and THP-1) and a mouse cell line (P388D1) were immunoprecipitated with anti-hAgo2 (4G8). Proteins were analyzed by SDS-PAGE and subjected to silver staining or western blotting with anti-hAgo2 (4G8). Half of the immunoprecipitated protein prepared from 1 × 10^7^ cells was loaded onto an SDS-polyacrylamide gel. IgG h.c. and l.c. indicate heavy chain and light chain of the antibody molecule, respectively. B: Recovery of small RNA from immunoprecipitates. Immunoprecipitated small RNAs were analyzed by Urea-PAGE and silver stained. The RNA fraction prepared from half of the immunoprecipitate from 1 × 10^7^ cells was loaded onto Urea-polyacrylamide gel. [file 1756-0500-2-169-S1.ppt]

## Slide 1
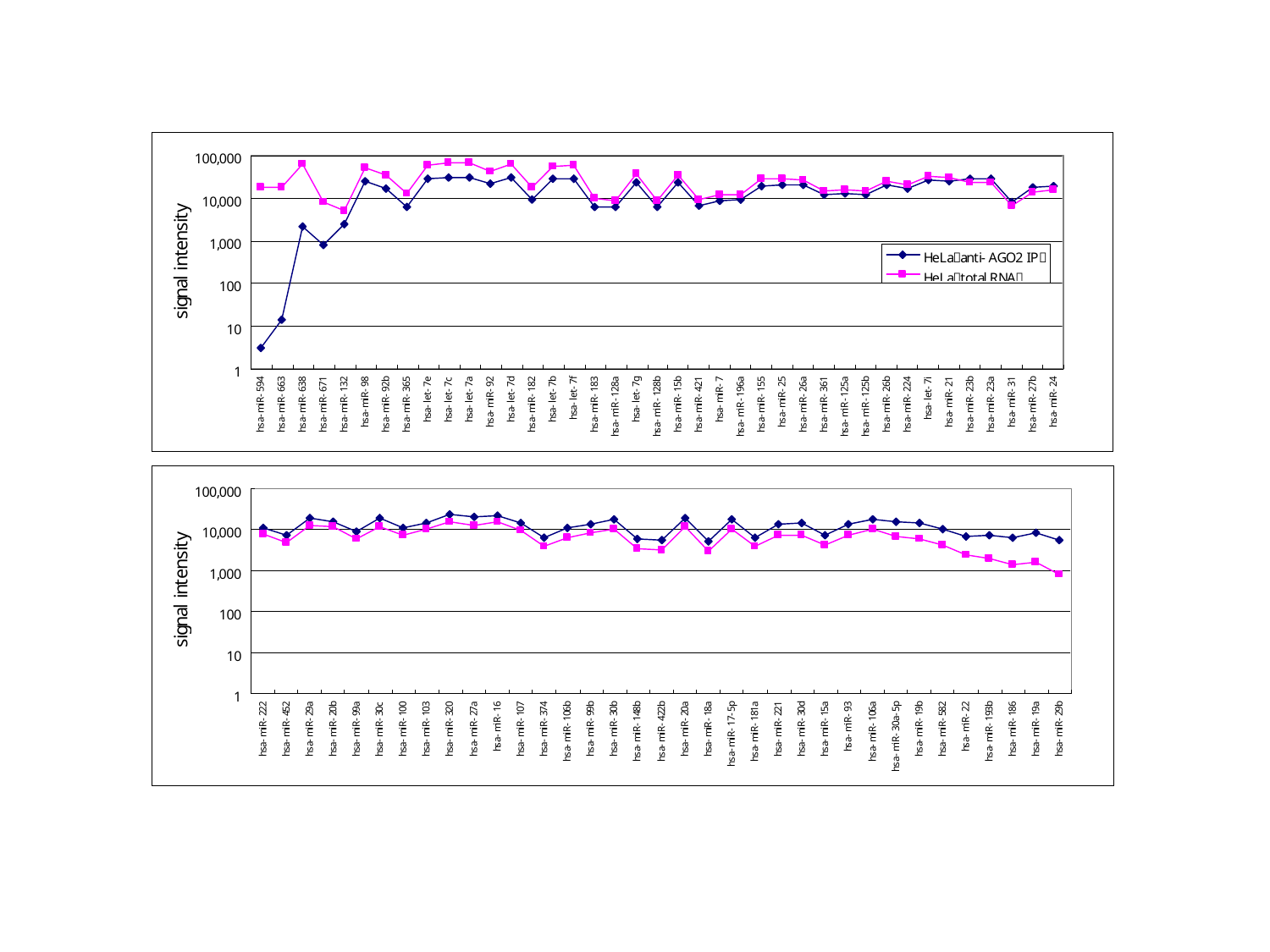

Supplement: Additional file 2 — Comparison of miRNA populations of total RNA and immunoprecipitated RNA in HeLa. Microarray analysis of miRNA was carried out at LC science inc. Immunopurified RNA and total RNA from HeLa cells were labeled with Cy3 and Cy5. Samples were hybridized to μParaflo® microfluidics chip with each of the detection probes containing a nucleotide sequence of coding segment complementary to human 474 microRNA sequences(miRBase ver.9.0). [file 1756-0500-2-169-S2.ppt]

## Slide 1
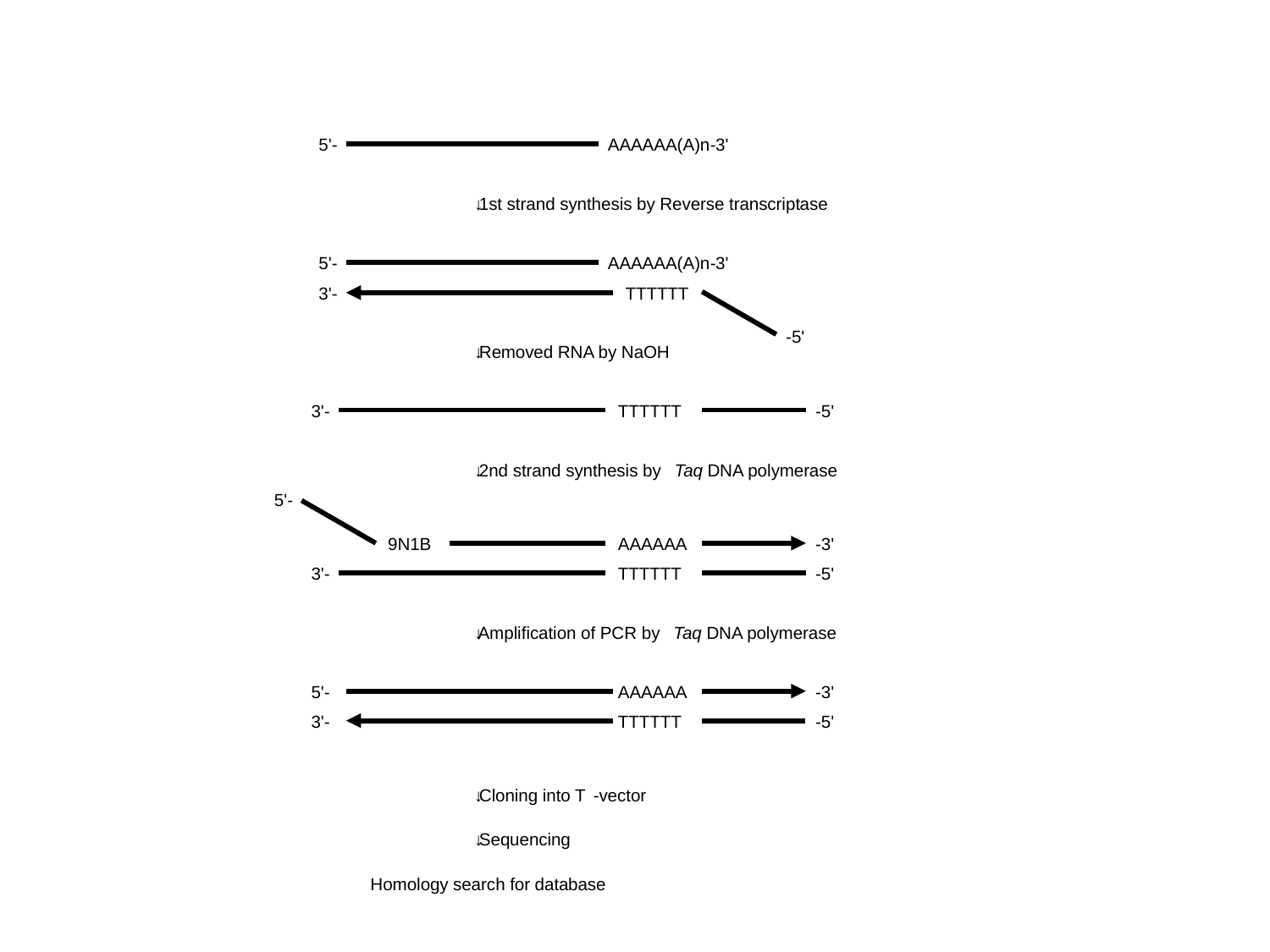

Supplement: Additional file 3 — Schematic representation of the cloning protocol for immunoprecipitated mRNA. The protocol is described in detail in Materials and methods (Additional file 1). [file 1756-0500-2-169-S3.ppt]
